# Genome-wide association studies on leaf midrib architecture in maize

**DOI:** 10.3389/fpls.2025.1671460

**Published:** 2025-10-30

**Authors:** Dongdong Dang, Shuwen Ji, Yubo Liu, Chunming Bai, Yanshu Zhu, Xuecai Zhang, Ao Zhang, Yanye Ruan

**Affiliations:** ^1^ Shenyang City Key Laboratory of Maize Genomic Selection Breeding, College of Bioscience and Biotechnology, Shenyang Agricultural University, Shenyang, Liaoning, China; ^2^ International Maize and Wheat Improvement Center (CIMMYT), El Batan, Texcoco, Mexico; ^3^ Seed Industry Innovation Research Institute, Liaoning Academy of Agricultural Sciences, Shenyang, Liaoning, China

**Keywords:** leaf venation, leaf vein, vascular bundle, single nucleotide polymorphisms (SNPs), YABBY gene

## Abstract

**Introduction:**

Leaf midrib architecture (LMA) in maize plays essential roles in supporting leaf structure and facilitating photosynthetic performance. Despite its importance for plant architecture and yield potential, the genetic basis underlying key midrib traits remains poorly understood.

**Methods:**

To dissect the genetic architecture of LMA, we performed a genome-wide association study (GWAS) using a diverse panel of 508 maize inbred lines representing tropical, subtropical, and temperate germplasm. Three traits—midrib width (MW), total midrib thickness (TMT), and midrib basal thickness (MBT)—were measured across two environments in Liaoning Province, China. Phenotypic data were analyzed using mixed linear models with population structure and kinship correction. Significant loci were identified under a Bonferroni-adjusted threshold, and candidate genes were functionally annotated and examined for expression patterns using publicly available transcriptomic data.

**Results:**

All LMA traits exhibited continuous variation and moderate heritability (H^2 = 0.33–0.61), with significant positive correlations among them. GWAS identified six SNPs significantly associated with LMA on chromosomes 5, 7, and 8, corresponding to 97 genes in adjacent genomic intervals. Among these, 27 annotated genes were enriched in functions related to transcriptional regulation, hormone signaling, cytoskeleton organization, and cell development. Key candidate genes included GRMZM2G074124 (YABBY-domain factor), GRMZM2G130953 and GRMZM2G332390 (auxin-related), GRMZM2G079185 (LOB-domain protein), and GRMZM2G407517 (Actin7). Correlation analyses further revealed that LMA traits are significantly associated with yield-related parameters, including cob diameter and grain weight.

**Discussion:**

Our findings demonstrate that LMA in maize is a complex quantitative trait governed by multiple genes with diverse biological functions. Several candidate genes involved in auxin response, cytoskeletal dynamics, and lateral organ development play crucial roles in midrib formation. The observed associations between midrib traits and yield components suggest that LMA can serve as a valuable target for improving canopy structure and productivity in maize breeding. These results provide novel insights and candidate loci for molecular dissection and genetic improvement of leaf midrib traits.

## Introduction

Leaf is a photosynthetic organ of plants which provides organic nutrients for whole plant (Sobrado and Turner, 1986; Abadie et al., 2018). The leaf photosynthetic cells and the other parts of plant are connected by vascular bundle system, on which the export of leaf photosynthetic products and input of nutrients and water needed by photosynthesis depend (Bagniewska-Zadworna et al., 2012; Dumlao et al., 2012). Leaf vascular system or venation also control the angle of leaf facing sunlight, which affects photosynthetic efficiency. A midrib is a midvein or primary vein locating at the longitudinal central axis of leaf blade, playing a pivotal role in the transport of substances and in the control on leaf upright angle and canopy architecture (Rolland-Lagan and Prusinkiewicz, 2005). However, the molecular mechanism of venation formation in maize remains unclear.

The development of venation starts at the leaf primordium, which is also the first step of leaf development. DL (drooping leaf) gene is essential for the process of venation development (Eshed et al., 2004). Loss-of-function mutations in the *dl* locus in rice cause lack of midrib, resulting in the drooping leaf phenotype (Ishikawa et al., 2009). DL gene is expressed in the central region of leaf primordia where the midrib develops subsequently and regulates cell proliferation (Yamaguchi et al., 2004). For Arabidopsis and rice, the DL gene encodes a zinc finger protein belonging to YABBY family of transcription factor, a plant-specific transcription factor family, which play an essential role in plant developmental processes by regulating the expression of many downstream genes, either directly or indirectly (Ohmori et al., 2008). The function of leaf midrib depends on its architecture such as thickness, width and concavity, but, the molecular mechanism that regulates leaf midrib architecture (LMA) is yet to be revealed, which may involve multiple quantitative genes.

Genome-wide association analysis (GWAS) is a powerful approach to identify the quantitative genes, via detecting the statistical association of a specific trait with genetic variants across multiple genomes (Visscher et al., 2017; Luo et al., 2024). Maize has enormous genetic diversity and rapid linkage disequilibrium decay, which is quite suitable for performing GWAS. Several quantitative traits in maize have been dissected by GWAS (Sahito et al., 2024), including low-phosphorus stress tolerance (Xu et al., 2018), field grain drying rate (Dai et al., 2017), husk number and weight (Zhou et al., 2016), aflatoxin accumulation resistance (Tang et al., 2015), water-stressed resistance (Xue et al., 2013), ear rot resistance caused by Fusarium verticillioides (de Jong et al., 2018).

In this study, 508 maize inbred lines with 558,630 high-quality single nucleotide polymorphism (SNP) marker sites were used to perform GWAS on three leaf midrib traits: width, total thickness and base thickness from two locations. Six significant SNPs have been located and 27 candidate genes, encoding many different families of proteins, are selected. These results show that the architecture of leaf midrib in maize is a quantitative trait regulated by multiple genes with different functions.

## Materials and methods

### Plant materials and field experiments

An association population of 508 maize inbred lines consist of germplasms collected from tropical, subtropical and temperate areas (Yang et al., 2011, 2013). There were 252 tropical/subtropical lines and 256 temperate lines (**Supplementary Table S1**). The association panel can also be classified into four subgroups (Yang et al., 2011): stiff stalk (SS), non-stiff stalk (NSS), tropical/subtropical (TST) and admixed group (MIX), respectively, with 27, 70, 196 and 215 inbred lines.

The population was grown under two environments in China: Fushun (N41°51′, E123°54′) and Tieling (N42°18′, E123°51′) of Liaoning Province in 2017. The experiments used a randomized complete block design with three blocks, row lengths of 2 m, row spacing of 0.6 m, and plant spacing of 0.3 m. The experiment was conducted in three replicates. Agronomic practices was identical in both environments.

### Phenotyping of midrib architecture

**Figure 1:**
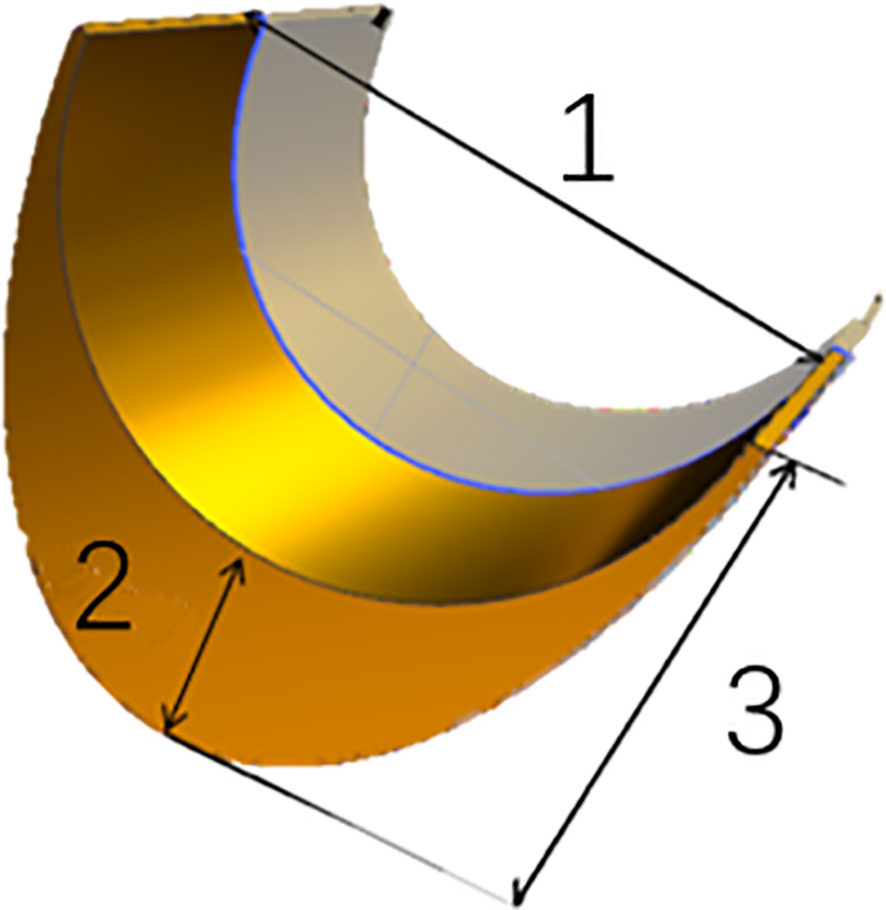
Architectural traits of leaf midrib in maize. 1 midrib width (MW); 2 midrib base thickness (MBT); 3 total midrib thickness (TMT).

Three traits of leaf midrib architecture (LMA): midrib width (MW), total midrib thickness (TMT) and midrib base thickness (MBT) were measured at the one third from the blade base of ear-leaf (**Figure 1**), at the same time during the maturity stage, using Electronic Vernier Caliper. More than five well-pollinated plants were selected from each line, respectively, in two environments.

### Phenotypic data statistical analyses

Correlation analysis, mapping, and analysis of variance for midrib architecture traits were carried out with TASSEL employing a mixed linear model (MLM). To avoid the occurrence of false-positive results, the MLM incorporated the influence of population structure (Q matrix) and affinities (K matrix), turning into K + Q model of MLM.

The MLM model algorithm is $y_{ijk}=\mu +Gen_{i}+Env_{j}+Gen_{i}\times Env_{j}+Rep_{k}+\epsilon_{ijk}$ Where $y_{ijk}$ is the trait of interest; μ  is the overall mean; $Gen_{i}$ , $Env_{j}$ , and are the effects of the *i-th* genotype, *j-th* year, and *i-th* genotype by *j-th* year interaction, respectively; is the effect of *k-th* replication; $\epsilon_{ijk}$ is the residual effect of the *i-th* genotype, *j-th* year, *k-th* replication.

Genotype is considered as the fixed effect, whereas all other terms are declared as the random effects. Locations with heritability below 0.05 were excluded from the across environment analysis. Best linear unbiased prediction (BLUP) was done by “PROC MIXED” in SAS software for estimation of leaf midrib trait values of all testers (Kump et al., 2011).

Phenotypic data were subjected to descriptive statistical analysis, analysis of variance, and correlation analysis using SPSS 19.0 (IBM Corp., Armonk, NY, USA). Broad-sense heritability was calculated using the formula proposed by Knapp et al. (1985): $h^{2}=\sigma_{g}^{2}/\left(\sigma_{g}^{2}+\frac{\sigma_{ge}^{2}}{nEnvs}+\frac{\sigma^{2}}{nEnvs\times nreps}\right)$ , Where $\sigma_{g}^{2}$ , $\sigma^{2}$ and are the genotypic variance, error variance, and genotype-by-environment interaction variance, respectively; and *nreps* and *nEnvs* are the numbers of replications and environments, respectively.

### Genome-wide association analysis

The BLUPs for each trait per inbred line across environments were estimated using PROC MIXED. All 508 lines were genotyped using the MaizeSNP50 BeadChip. The genome-wide association analyses based on these models were conducted with the software in the FarmCPU procedure in R-4.2.1 (Liu et al., 2016). We conducted genome-wide association mapping with 558,630 SNPs (MAF > 0.05) on phenotypic data of each environment. A Bonferroni-corrected threshold probability based on individual tests was calculated to correct for multiple comparisons, using P< 1/N, where N is the number of individual trait-SNP combinations tested, the final threshold is set at P<1.04 × 10^-5^.

The independent contribution rates of significant SNP loci to midrib traits were determined based on the Bonferroni-corrected threshold and were defined as the phenotypic contribution rates. These calculations were performed using the anova function in R. The algorithm is as follows:


$$Y=\alpha \sum_{i=1}^{n}X_{i}+\beta P+\epsilon$$

Where Y represents the three variables of leaf midrib traits; X represents the SNP genotype variable corresponding to each trait; P is the subgroup variable; i is the number of individuals in the population; α is the SNP effect; β is the subgroup effect; and ϵ is the random error term.

### Gene function prediction

We used the maize line B73 reference genome (B73 RefGen_v2, https://www.maizegdb.org/) to identify candidate genes that were either included or close to the significantly associated SNPs. Annotated functions and relevant information for the candidate genes were obtained from the Maize Genetics and Genomics Database and the U.S. National Center for Biotechnology Information (http://www.ncbi.nlm.nih.gov/). Finally, possible candidate genes were selected based on the functional annotations of all genes in the SNP locus and their expression in each tissue of B73.

### Heat map of candidate genes

**Figure 2:**
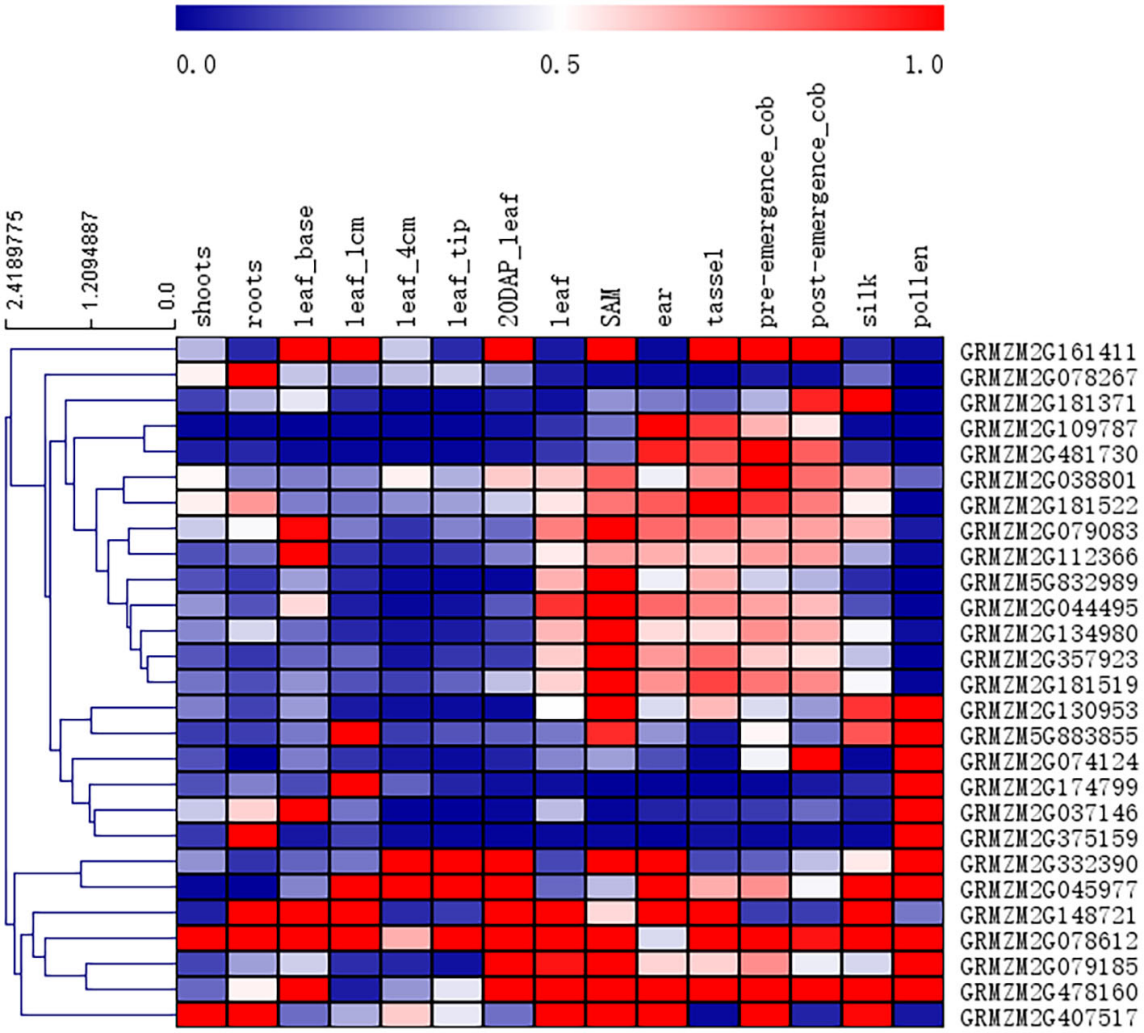
Heat map of tissue-specific expression patterns of candidate genes identified by GWAS. The value used in the figure is the PRKM counts in leaf tissue and other tissues, as shown at the bottom of each column. Columns and rows are sorted according to similarity (top and left are hierarchical cluster analysis). Red, white and blue indicate the degree of expression in different tissues.

The raw RNA-Seq data, originating from Genevieve M. Hoopes et al. and downloaded from the NCBI database, were aligned to the maize B73 reference genome (RefGen_v2), and the corresponding raw transcriptomic reads are available from NCBI under the following BioProject IDs: PRJNA171684, PRJEB10574, PRJNA226757, PRJNA244661, PRJNA323555, and PRJNA369690 (Hoopes et al., 2019)., and the RNA-Seq reading aligned with the maize B73 reference genome (RefGen_v2). The Cufflinks software keeps only unique map reads for counting FPKMS. The value used in **Figure 2** is the normalized FPKM count in the pericarp with respect to other tissues.

## Results

### Trait variation and heritability

Under the two environments, the MW range of the association mapping population is 2.99-11.34mm, the TMT range is 0.99-7.28mm, and the MBT range is 0.23-4.42mm, and the ranges of trait variations are wide (**Table 1**). Significant variance components for genotype (G) and genotype×environment (G×E) interactions were observed for all three traits, however, G×E interactions represented relatively a small proportion of the total variance. According to the formula proposed by Knapp et al. (1985). The broad-sense heritability’s (H^2^) estimated for MW, TMT, and MBT were moderate, which were 0.59, 0.61, and 0.33, respectively. The H^2^ of midrib traits estimated show that leaf midrib architecture traits are controlled by genetic effects, thus these traits are suitable for the association analysis.

**Table 1:** Variance composition and broad-sense heritability of leaf midrib architecture traits in the association population of maize.

Traita	BLUPs Means ± SD(mm)^e^	Range (mm)^e^	Skewness^e^	Kurtosis^e^	Variance component^b,c^			H^2^ ^d^
					G	E	G × E	
MW	6.65 ± 0.55	2.99-11.34	-0.54	-3.2	997.75**	207.41*	312.11*	0.59
TWT	3.69 ± 0.387	0.99-7.28	0.16	-1.67	516.18**	78.28*	152.40*	0.61
MBT	1.79 ± 0.19	0.23-4.42	-0.04	-1.73	157.08**	74.62*	72.47*	0.33

a MW, midrib width; TMT, total midrib thickness; MBT, midrib base thickness.

b G and E indicate genotype and environment, respectively, and G × E indicate interaction of G and E.

c *Significant at *P* ≤ 0.05; **Significant at *P* ≤ 0.01.

d Family mean-based broad-sense heritability.

e BLUPs Mean ± SD, Range, Skewness, and Kurtosis are computed from genotype-level BLUPs aggregated across environments.

**Figure 3:**
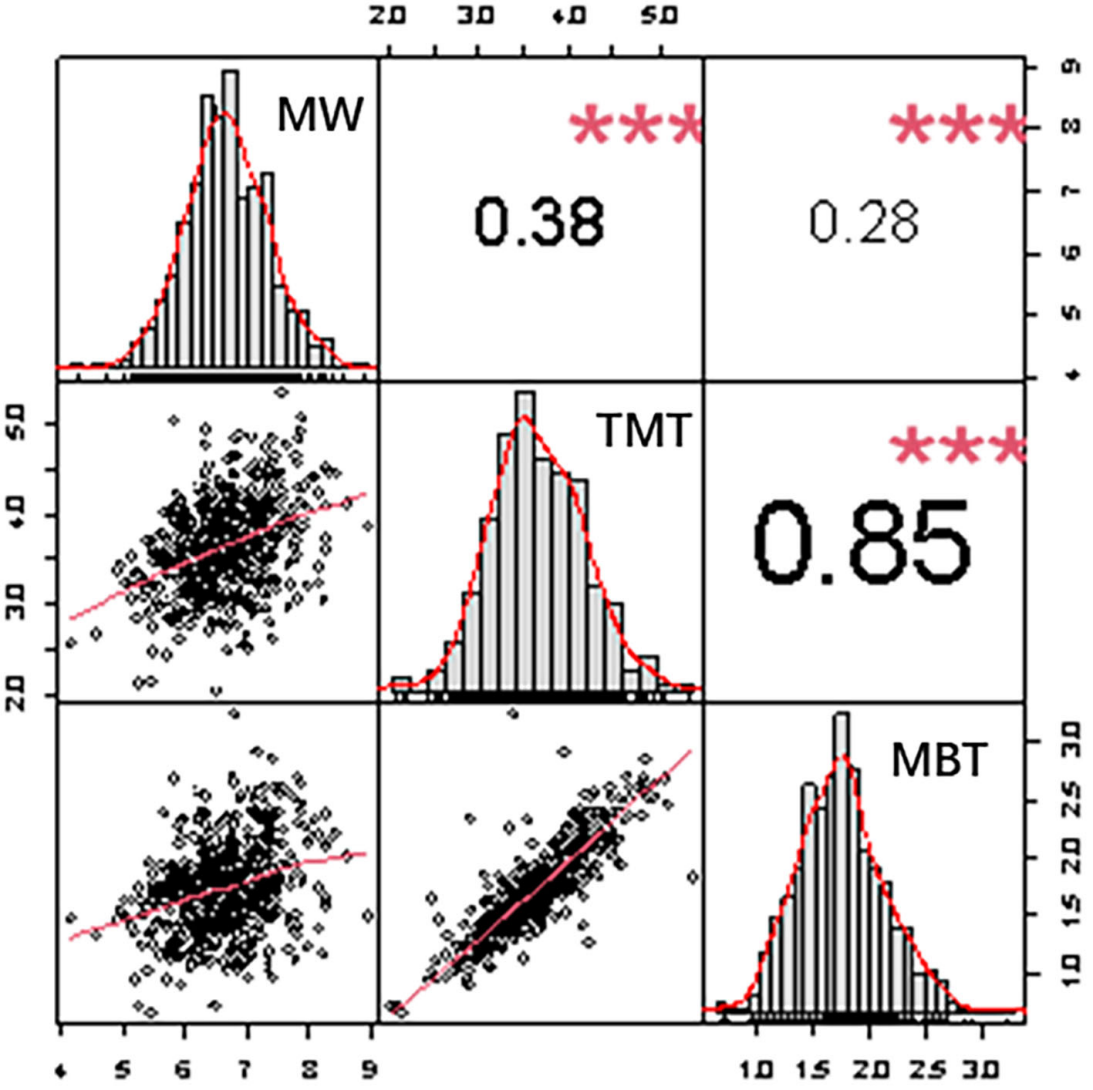
Frequency distributions and correlations of three leaf midrib architecture traits. Plots on diagonal line show phenotypic distribution of each trait as indicated; values above diagonal line are Pearson’s correlation coefficients between traits; plots below diagonal line are scatter plots of compared traits. MW, midrib width; TMT, total midrib thickness; MBT, midrib base thickness. * Represents the significance level calculated at P ≤0.05; ** represents the significance level calculated at P ≤ 0.01.

The best unbiased linear predictive value (BLUP) of three midrib traits shows a normal distribution, and the correlation between them are significant positive correlation (p ≤ 0.01) (**Figure 3**). TMT is highly positive correlated with MBT (r = 0.85, p ≤ 0.01), Pearson correlation coefficient reaching 0.87, the thicker the midrib thickness is, the thicker the midrib base thickness is. The significant correlation is present between leaf midrib architecture traits, indicating that the growth and development of LMA traits are highly coordinated.

**Figure 4:**
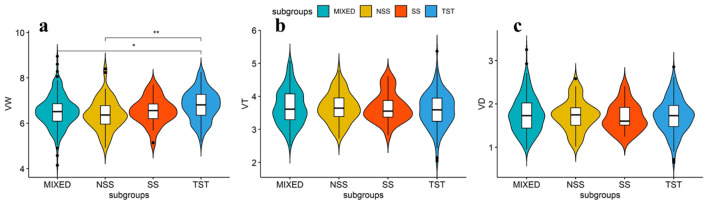
Boxplots of the distribution of leaf midrib architecture traits in four subpopulations. Analysis of variance (ANOVA) was applied to examine the difference of traits among subpopulations. Different numbers indicate statistically significant difference at P ≤ 0.05. No. of inbred lines included in each subpopulation are 215, 70, 27 and 196 for MIXED (admixed), NSS (non-stiff stalk), SS (stiff stalk) and TST (tropical/subtropical), respectively. **(a)** MW (midrib width); **(b)** TMT (total midrib thickness); **(c)** MBT (midrib base thickness).

The four subgroups, SS, NSS, TST and MIXED, of the maize association panel have different origins (Yang et al., 2011). The SS and NSS subpopulations are of temperate origins, TST subpopulations is of tropical and subtropical origins, and MIXED subpopulations contain other inbred lines that are not classified in the afore mentioned three subpopulations (Yang et al., 2011). We compared the variation of leaf midrib architecture traits among different subgroups through boxplots (**Figure 4**). The mean value of MW in the TST subgroup was larger compared with SS, NSS and MIXED subgroups, indicating that for the tropical/subtropical origins inbred lines of maize, MW tends to be wider (**Figure 4A**). For TMT and MBT, no significant differences in the mean value were observed between the subgroups, indicating that the population origins has no significant effect on leaf midrib traits of thickness (**Figures 4B, C**). However, the difference of leaf midrib architecture traits between inbred lines in each subgroup were observed (**Figure 4**), which endows the possibility of genetic improvements on LMA.

### Leaf midrib architecture traits related to other agronomic traits

**Figure 5:**
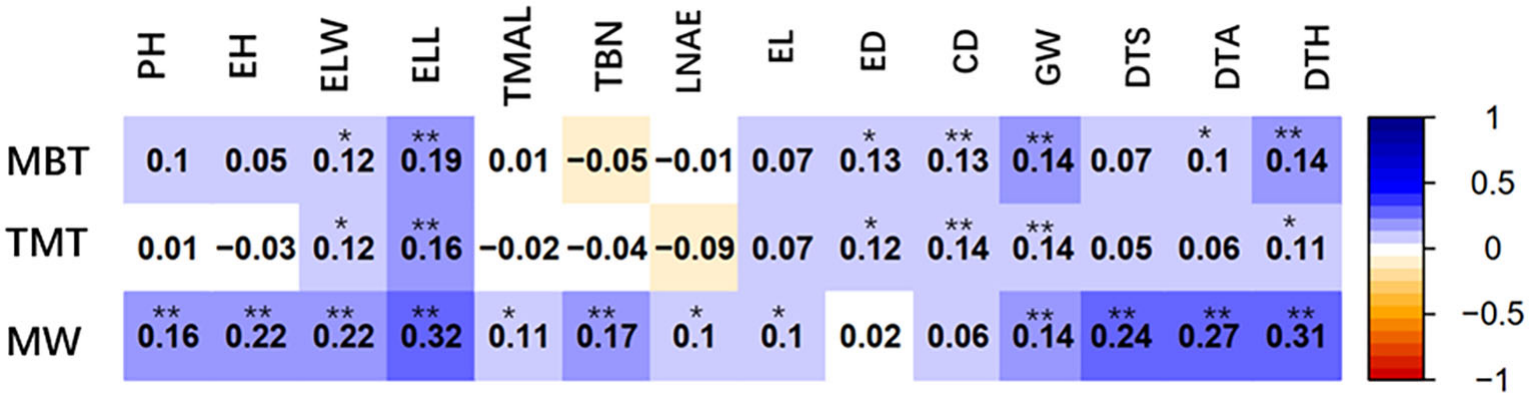
Correlation coefficient between leaf midrib architecture traits and other 14 agronomic traits based on BLUP value. MW, midrib width; TMT, total midrib thickness; MBT, midrib base thickness. PH, plant height; EH, ear (position) height; ELW, ear leaf width; ELL, ear leaf length; TMAL, tassel maximum axis length; TBN, tassel branch number; LNAE, leaf number above ear; EL, ear length; ED, ear diameter; CD, cob diameter; GW, cob grain weight; DTA, days to anthesis; DTS, days to silking; DTH, days to heading. *Significant at P ≤ 0.05; **significant at P ≤ 0.01.

In this study, correlation analysis was conducted on the width, total thickness and base thickness of leaf midrib architecture with 14 agronomic traits, including 7 plant traits, plant height (PH), ear (position) height (EH), ear leaf width (ELW), ear leaf length (ELL), tassel maximum axis length (TMAL), tassel branch number (TBN), leaf number above ear (LNAE); and 4 ear and kernel traits, ear length (EL), ear diameter (ED), cob diameter (CD), cob grain weight (GW); and 3 fertility stage traits, days to anthesis (DTA), days to silking (DTS) and days to heading (DTH). As shown in **Figure 5**, the three leaf midrib architecture traits are positively and significantly correlated with two their own ear leaf blade traits of ELW and ELL, indicating that the ear leaf blade traits and midrib architecture traits develop synchronously and interplay each other. The midrib traits are also significantly correlated with GW and other grain traits. The MW is significantly correlated with DTS, DTA and DTH.

### GWAS for leaf midrib architecture traits

**Figure 6:**
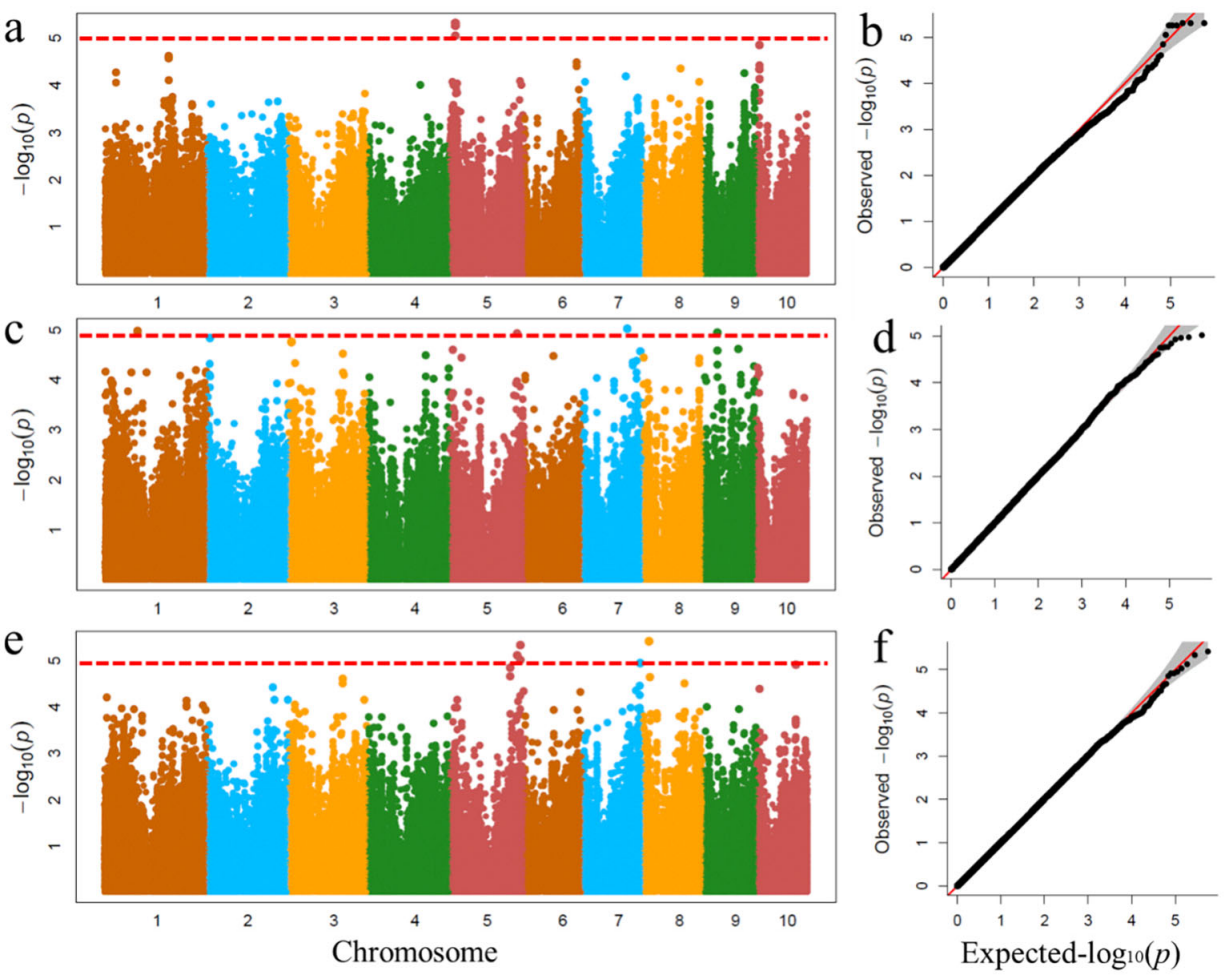
Genome-wide association studies of leaf midrib architecture traits. GWAS-derived Manhattan plots and Quantile-quantile plot showing significant P-values associated with leaf midrib architecture traits using MLM. Each dot represents an SNP. The horizontal dashed red line represents the Bonferroni-corrected significant threshold 1.04 × 10^-5^. **(a, b)** MW; **(c, d)** TMT; **(e, f)** MBT.

Genome-wide association analyses were performed using the FarmCPU procedure implemented in R. To minimize the influence of environmental variation, phenotypic BLUP values from two environments were used for GWAS (**Figure 6**). The results showed that four significant SNPs associated with MBT were identified on chromosomes 5 and 8, one significant SNP associated with TMT was detected on chromosome 7, and one significant SNP associated with MW was identified on chromosome 5.

### Genes co-localized with significant GWAS SNPs

Searching the range of 50 kb for each significant SNP upstream and downstream, a total of 97 genes (**Supplementary Table S2**) were found in the afore-mentioned 6 sites, based on the B73 reference genome and the LD attenuation distance of each significant site. Of which, 31 genes have functional annotations, and the functions of the remaining 66 genes are unknown. In detail, for the MBT trait, 30 genes were located, of which 12 are annotated; for the TMT, 17 genes were determined, among them 3 are annotated; for the MW, up to 50 genes were detected, 12 of which are annotated (**Supplementary Table S3**).

To determine whether these candidate genes are specifically expressed in leaf venation traits, the published RNA-seq data set were used to compile expression patterns in different organs/tissues, and a heat map of the tissue-specific expression patterns of candidate genes was produced (**Figure 2**). Twenty-seven annotated genes were identified as candidate genes associated with the leaf midrib architecture traits. According to NCBI and previous research results, these genes were divided into 6 categories, including metabolism, cell transport, transcriptional regulation, plant hormone regulation, structural proteins, and cell division, which directly or indirectly regulate leaf and midrib architecture trait development in maize.

## Discussion

The ultimate goal of maize breeding is not only to improve the quality of grains, but more importantly to enhance the grain yield. The realization of these goals depends on the excellent overall agronomic traits of crop plants. Leaf architecture directly influences canopy structure, consequentially affecting yield. Therefore, it is necessary to clarify the relationship between quality traits, agronomic traits and yield traits. Leaf midrib is responsible for the mutual transport of nutrients and water between the leaf and stalk of plant. It also provides mechanical support to leaf blades and controls the angle of leaf facing sunlight, which influences the efficiency of leaf photosynthesis. The correlation analysis suggests that the ear leaf midrib architecture traits develop synchronously with leaf blade traits and interplay each other; the midrib traits are also significantly correlated with GW and other grain formation traits (**Figure 5**). The transport capacity and rigidity of leaf midrib are affected by its architecture. Therefore, defining the suitable midrib architecture and dissecting its genetic basis is very important for ideal crop architecture breeding by molecular marker assisted selection.

### Leaf midrib architecture is genetically controlled

Current researches have shown that leaf venation is a quantitative trait under polygenic control, with high heritability (Al-Shugeairy, 2016; Rishmawi et al., 2017). In this study, the LMA traits of 508 maize inbred lines manifest continuous distribution, and high broad-sense heritability (**Table 1**), indicating that the three midrib architecture traits in this panel are conditioned mainly by genetic factors. The value of MBT is stable in two environments, which indicates that the trait has good recurrence in different environments and further demonstrates that it is feasible to map genes related to leaf midrib architecture traits. The larger mean width of leaf midrib appears in the tropical/subtropical origins inbred lines (**Figure 4**), suggesting that this subgroup possesses abundant or special genes to promote the leaf midrib width development. Although the mean values of TMT and MBT do not show significant difference among subgroups, their significant variations are observed in one single subgroup. Therefore, it is possible to modify leaf midrib architecture using genetic resources inside the subgroups of maize.

### Significant correlation of leaf midrib architecture with reproduction traits

The leaf midrib architecture traits are not only significantly correlated with the length (ELL) and width (ELW) of their own ear leaf blade, but also with the reproduction traits, including ear length (EL), ear diameter (ED), cob diameter (CD), cob grain weight (GW), days to anthesis (DTA), days to silking (DTS) and days to heading (DTH) (**Figure 5**). These correlations show that well-developed leaf midrib architecture traits facilitate the reproductive process in maize. The leaf midrib architecture is a potential aspect of improving whole plant architecture and grain production in maize.

### Leaf midrib architecture is regulated by quantitative genes

The leaf midrib architecture is identified as quantitative traits by heritability and phenotypic variation in the population (**Table 1**; **Figure 4**). The MBT trait is associated with 4 significant SNPs, the TMT and MW traits are only associated with 1 significant SNP, respectively. However, this does not mean that the TMT and MW traits are not controlled by multiple genes, because many different relative genes may locate in the adjacent region of significant SNP. It has been reported that some similar function or function-related genes locate closely each other on chromosome (Calusinska et al., 2015; Wu et al., 2021). In this study, we screened out several candidate genes regulating LMA traits from the adjacent region of each significant SNP locus (**Figure 6**), their expression supports their functions in LMA trait development (**Figure 2**). Twenty-seven from 97 genes identified in 6 significant SNP loci associating LMA are functionally annotated (**Figure 2**). These candidate genes may participate in modifying gene transcription, regulating auxin functions, small RNA transcription and function, cytoskeleton, cell division and movement, lateral organ growth.

The candidate gene *GRMZM2G074124* is associated with MW, presumed to be a YABBY domain transcription factor family protein. YABBY gene family play important roles in the growth and polar establishment of lateral organs such as leaves and floral organs in plants (İnal et al., 2017). The loss-of-function mutations of *dl* gene belonging to this family cause lack of midrib and the drooping leaf phenotype (Ishikawa et al., 2009) (Yamada et al., 2011). In maize, pleiotropic mutations in drl1 was reported to affect leaf length and width, leaf angle (Strable et al., 2017). This study adds a new function of YABBY domain transcription factor to regulate leaf midrib architecture.

*GRMZM2G130953*, an associated gene with MBT, encodes IAA27 protein, an auxin-response transcription factor, and *GRMZM2G332390* is located in the significant SNP locus of MW, encoding ZmSAUR (SMALL AUXIN UP RNA) 48 protein (Chen et al., 2014), both belonging to early auxin response genes (Hagen and Guilfoyle, 2002). IAA27 protein can regulate the activity of ARF transcription factors (Galli et al., 2015) and SAUR gene is mainly expressed in the hypocotyl and other elongating tissues (Stamm and Kumar, 2013; Favero et al., 2016; Spartz et al., 2017). Therefore, GRMZM2G130953 and GRMZM2G332390 may participate in the development of leaf midrib width and base thickness…. which binds to calmodulin in a calcium-dependent manner, suggesting that they may mediate responses to changes in intracellular calcium level (Yang and Poovaiah, 2000).

*GRMZM2G109787* and *GRMZM2G481730*, both associated with MBT, encode RNA-dependent RNA polymerases (RDR) to synthesize sRNAs (Qian et al., 2011; Kelliher and Walbot, 2014). The two main types of small RNAs are microRNAs (miRNAs) and small interfering RNAs (siRNAs) (Carthew and Sontheimer, 2009). *GRMZM2G045977*, also associated with MBT, is a target gene of miR396, a growth-regulating factor for maize (Liu et al., 2014), suggesting that miR396 regulates midrib formation (Debernardi et al., 2012).

The candidate gene *GRMZM2G078612*, associated with MW, encodes γ tubulin -2 chain; *GRMZM5G832989*, associated with MBT, encodes microtubule-associated protein 70-1. Microtubule (MT) is an important component of cytoskeleton. *GRMZM2G407517*, associated with MBT, encodes Actin7 is involved in gibberellin and brassinosteroid biosynthesis, auxin transport and cellulose synthesis (Wei et al., 2018). *GRMZM2G079185*, associated with MW, encodes the LOB (lateral organ boundaries) domain protein, which regulate lateral organ growth in plants (Zhang et al., 2014; Gombos et al., 2017).

## Conclusion

The leaf midrib architecture traits width (MW), total thickness (TMT) and base thickness (MBT) have higher heredity and are controlled by quantitative genes in maize. These leaf traits are also significant correlated with reproduction traits such as days to anthesis (DTA), days to silking (DTS), days to heading (DTH), ear length (EL), ear diameter (ED), cob diameter (CD), and cob grain weight (GW). Six SNP loci are significantly associated with LMA, and 97 genes are identified in the SNP location interval.

## Data Availability

The original contributions presented in the study are included in the article/**Supplementary Material**. Further inquiries can be directed to the corresponding authors.

## Supplementary material

Supplementary Table 1 Genome-wide diversity and haplotype summary of maize inbred lines.

Supplementary Table 2 The candidate gene information.

Supplementary Table 3 Information of candidate genes for leaf midrib architecture in maize. ^a^candidate genes were found in maizegdb. MW, midrib width; TMT, total midrib thickness; MBT, midrib base thickness.
